# IHCP: interpretable hepatitis C prediction system based on black-box machine learning models

**DOI:** 10.1186/s12859-023-05456-0

**Published:** 2023-09-06

**Authors:** Yongxian Fan, Xiqian Lu, Guicong Sun

**Affiliations:** School of Computer Science and Information Security, Guilin University of Electronic Technology, Guilin, 541004 China

**Keywords:** Hepatitis C, Machine learning, Interpretable artificial intelligence, SHAP, LIME

## Abstract

**Background:**

Hepatitis C is a prevalent disease that poses a high risk to the human liver. Early diagnosis of hepatitis C is crucial for treatment and prognosis. Therefore, developing an effective medical decision system is essential. In recent years, many computational methods have been proposed to identify hepatitis C patients. Although existing hepatitis prediction models have achieved good results in terms of accuracy, most of them are black-box models and cannot gain the trust of doctors and patients in clinical practice. As a result, this study aims to use various Machine Learning (ML) models to predict whether a patient has hepatitis C, while also using explainable models to elucidate the prediction process of the ML models, thus making the prediction process more transparent.

**Result:**

We conducted a study on the prediction of hepatitis C based on serological testing and provided comprehensive explanations for the prediction process. Throughout the experiment, we modeled the benchmark dataset, and evaluated model performance using fivefold cross-validation and independent testing experiments. After evaluating three types of black-box machine learning models, Random Forest (RF), Support Vector Machine (SVM), and AdaBoost, we adopted Bayesian-optimized RF as the classification algorithm. In terms of model interpretation, in addition to using common SHapley Additive exPlanations (SHAP) to provide global explanations for the model, we also utilized the Local Interpretable Model-Agnostic Explanations with stability (LIME_stabilitly) to provide local explanations for the model.

**Conclusion:**

Both the fivefold cross-validation and independent testing show that our proposed method significantly outperforms the state-of-the-art method. IHCP maintains excellent model interpretability while obtaining excellent predictive performance. This helps uncover potential predictive patterns of the model and enables clinicians to better understand the model's decision-making process.

## Background

The liver plays a vital role in many essential functions in the human body. Any damage to the liver will adversely affect critical physiological processes and the patient’s health status [1, 2]. At the same time, the early stages of liver disease are often difficult to diagnose, because even if partially infected, they do not affect the normal functioning of the liver. Moreover, in the case of depleted liver capacity, life can only last one or two days [3]. Therefore, early diagnosis of hepatitis is crucial for both doctors and patients [4]. Among them, hepatitis C is an inflammatory liver disease caused by the hepatitis C virus (HCV) and is the principal global cause of chronic hepatitis, hepatic sclerosis, and hepatocellular carcinoma [5, 6]. WHO estimates that around 290,000 people will die from hepatitis C in 2019, mainly from cirrhosis and hepatocellular carcinoma (primary liver cancer) [7]. By 2022, WHO reports that diagnosis and treatment of hepatitis C will be interrupted in half of the countries due to the COVID-19 pandemic [8]. Multiple studies have shown that early detection remains the best option to improve the survival rate of patients with liver disease [3, 9]. Therefore, exploring serum-based prediction methods for hepatitis C is important for the early detection and treatment of hepatitis.

In recent years, machine learning techniques are rapidly applied in different medical applications [10–13], such as chronic COVID-19, fatty liver disease, liver disease [14], kidney disease, heart disease, and diabetes. This technique uses large datasets and statistical methods to identify complex relationships between patient medical attributes and outcomes. The two main medical areas currently using machine learning are diagnosis and outcome prediction. In particular, machine learning is a valuable tool for identifying individuals at high risk of health deterioration.

A number of studies have used machine learning techniques to study hepatitis in the last few years [6, 14–17]. The application of machine learning methods has greatly improved the predictive performance of hepatitis, but the interpretation of the underlying predictors is generally lacking.

In this study, we introduce a combined method of IHCP to predict hepatitis C, which integrates an interpretable model based on SHAP and LIME_stabilitly with a machine learning method. IHCP combines interpretability with high predictive performance. More importantly, our interpretable model can help physicians identify hepatitis at an early stage and help cure patients at an early stage of hepatitis.

We summarize the contributions of this study as follows:

1. We propose a hepatitis C prediction method IHCP. IHCP introduces RF, AdaBoost, and SVM machine learning models for early hepatitis prediction, and uses SHAP and LIME_stability for interpretability analysis.
2. Comparative experiments based on the UCI dataset and an independent testing set show that IHCP significantly outperformed the current most advanced methods.
3. IHCP conducts interpretable analyses to validate the factors that have the greatest impact on the patient population and help healthcare providers to predict hepatitis at an early stage and prevent the deterioration of the disease.

## Result and discussion

### RF hyperparameter setting

Based on the experimental analysis, different datasets need to be trained with different hyperparameters in the Bayesian optimized RF, and the optimization parameters on the UCI dataset and the independent testing set are respectively recorded in Tables 1 and 2.

**Table 1:** RF model hyperparameters settings in the UCI dataset

Hyperparameter	Value
n_estimators	500
max_depth	70
max_features	2
min_samples_split	None
min_samples_leaf	None
max_leaf_nodes	None

**Table 2:** RF model hyperparameters settings in the independent testing set

Hyperparameter	Value
n_estimators	102
max_depth	100
max_features	1
min_samples_split	None
min_samples_leaf	None
max_leaf_nodes	None

### Performance evaluation of different machine learning algorithms

**Fig. 1:**
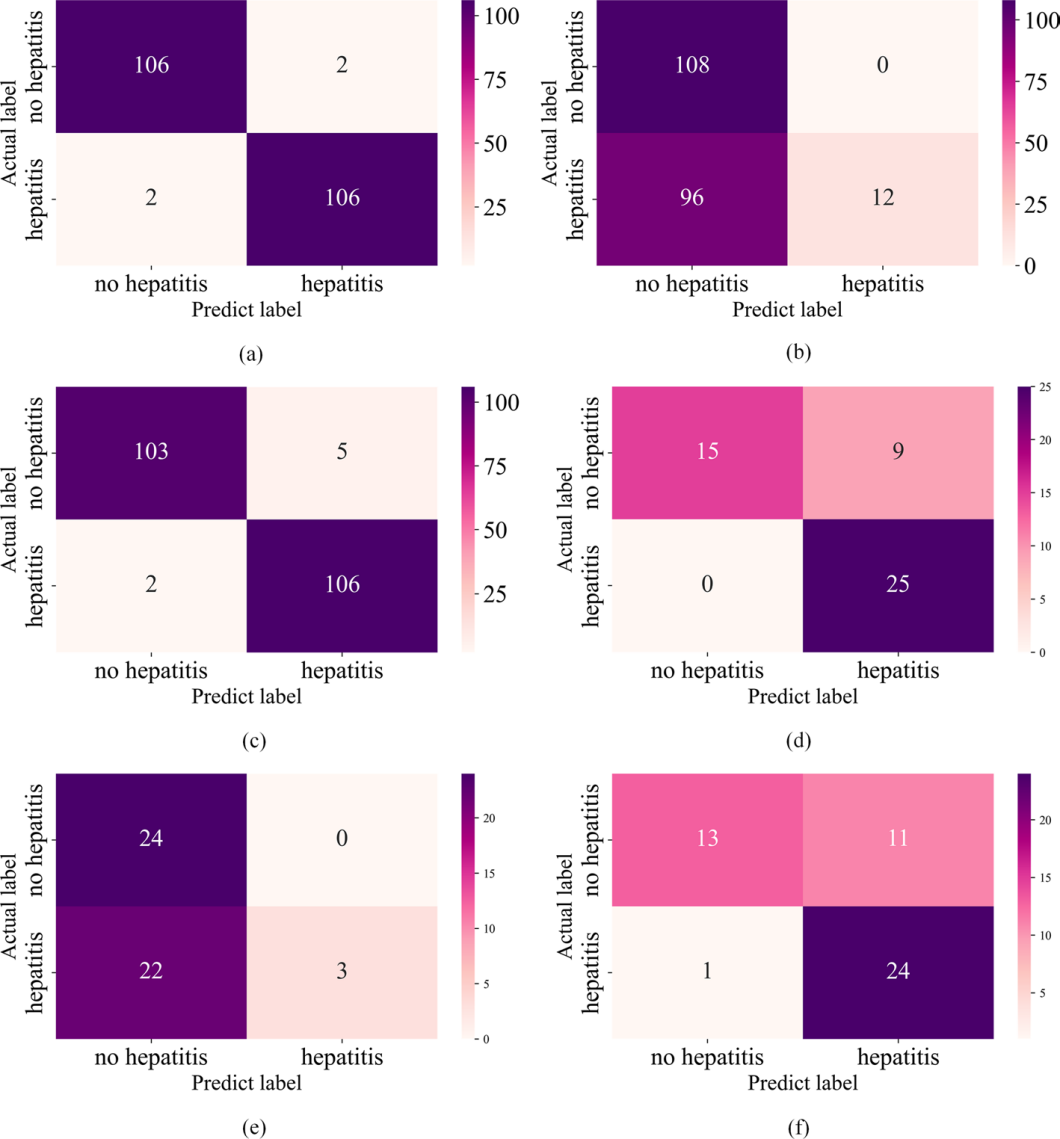
Confusion matrix. **a** Confusion matrix of RF in the UCI dataset. **b** Confusion matrix of SVM in the UCI dataset. **c** Confusion matrix of AdaBoost in the UCI dataset. **d** Confusion matrix of RF in the independent testing set. **e** Confusion matrix of SVM in the independent testing set. **f** Confusion matrix of AdaBoost in the independent testing set

In this section, we compare the performance of different machine learning algorithms in predicting hepatitis C patients using fivefold cross-validation. The confusion matrices of the RF, SVM, and AdaBoost models are shown in Fig. 1. As can be seen from the figure, SVM has the worst prediction results and will easily misdiagnose hepatitis C patients as blood donors. In clinical practice, it does not serve as an early diagnosis. The AdaBoost is most likely to diagnose blood donors as hepatitis C patients, which may cause unnecessary patient panic. In comparison, RF is the best-performing model among them.

Table 3 shows the performance comparison of the three classifiers in the UCI dataset. It can be seen that RF performed the best among all five evaluation indicators, it achieved a correct rate of 0.9944 and an AUC value of 0.9986. And Table 4 shows the comparison of the performance of the three classifiers in the independent testing set, and it can be seen that, overall, it is still RF that performs the best. Therefore, we choose to use Bayesian-optimized RF as the proposed classifier for hepatitis diagnosis.

**Table 3:** Comparison of the three machine learning classifiers in the UCI dataset

Classifier	Accuracy	AUC	Precision	Recall	F1-score
RF	**0.9944**	**0.9986**	**0.9946**	**0.9954**	**0.9944**
SVM	0.8546	0.9241	0.9821	0.7248	0.7684
AdaBoost	0.903	0.9514	0.9759	0.8319	0.8515

Bold represents the best performing indicator

**Table 4:** Comparison of the three machine learning classifiers in the independent testing set

Classifier	Accuracy	AUC	Precision	Recall	F1-score
RF	**0.9148**	**0.9895**	0.9008	**0.9277**	**0.9052**
SVM	0.7298	0.7662	**0.9504**	0.5207	0.5545
AdaBoost	0.771	0.8236	0.9136	0.6481	0.6571

Bold represents the best performing indicator

### Explainable models based on SHAP and LIME

Several recent studies have introduced new interpretable methods to explain the prediction process and underlying mechanisms of machine learning classifiers. Interpretable methods are divided into global and local interpretable techniques [18]. We extended our machine learning model by using SHAP [19] and LIME [20]. LIME is the most commonly used local interpretation method, and SHAP is the most popular global interpretable method. Global interpretability aims to help one understand the overall logic behind complex models and the internal working mechanism, and local interpretability aims to help one understand the decision process and decision basis of machine learning models for each input sample.

**Fig. 2:**
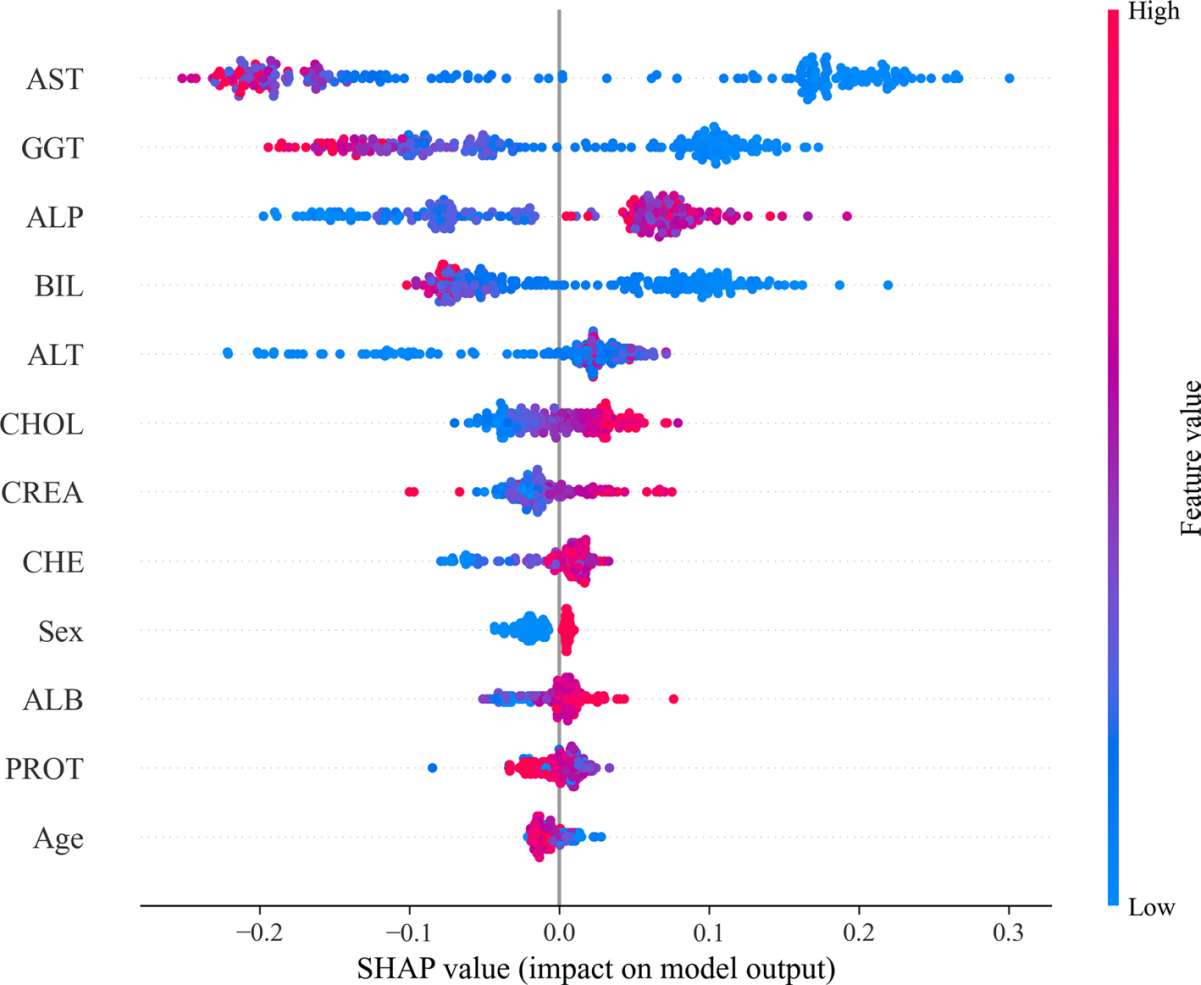
SHAP summary diagram

**Fig. 3:**
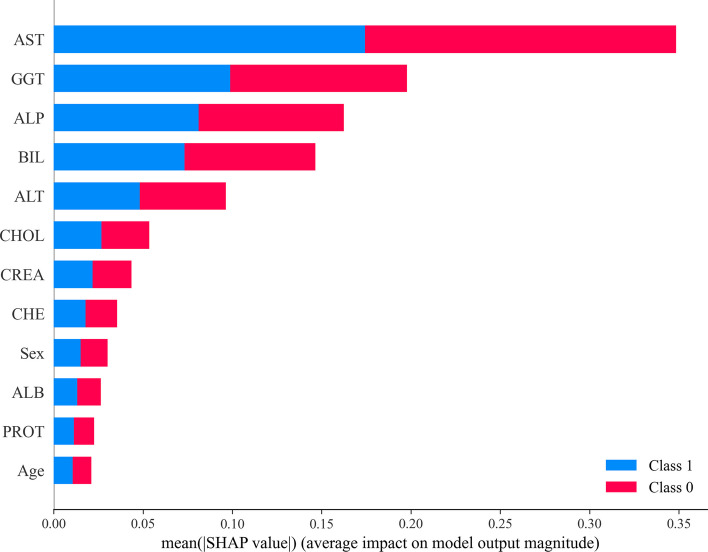
Summary of the average absolute SHAP values on the model targets

SHAP uses the SHAP value to measure the impact of the characteristics of a complex model. The SHAP value is defined as the weighted average of the marginal contributions [21]. It can be used to explain any type of predictive model for classification or regression [21]. Figure 2 shows a summary plot of the SHAP values of our proposed hepatitis diagnostic model. Where the horizontal coordinate is the SHAP value and the vertical coordinate is the feature type. Each point’s color determines the element's value; higher values are marked in red and lower values are marked in blue. The summary plot depicts the relationship between each feature and the final prediction of the model, the probability that the sample is malignant. All features are ranked on the y-axis according to their importance in the prediction. Figure 3 visualizes the binary output of the hepatitis diagnostic model. The visualization shows the mean absolute Shapley values of 12 characteristics for hepatitis patients and non-hepatitis patients, where the hepatitis patient category is represented by “1” and the no hepatitis patient category is represented by “0”. Based on the two visualizations, the most important features among the 12 visualized features are AST, GGT, ALP, BIL, and ALT, with AST having the highest priority. In contrast, some features, such as Age and PROT, have low priority in determining whether a patient has hepatitis.

LIME, a black-box model interpretation method, interprets the model by providing a model that behaves very similarly to the original model [20]. It approximates the black box model *f* by using a simple function g around a point* x*, where *g* must belong to the class of interpretable models *G*. Each model corresponds to a specific input point *x*, only around *x* are the predictions of the interpretable model guaranteed to be very close to the black box model. This property determines the ability of LIME to act as a local interpretable tool.

Each time LIME is used, it generates new data points that follow the same distribution but differ in different applications. Due to the random nature of sampling, using different issues, different interpretable models may be obtained, thus obtaining different interpretations for the selected individuals [22]. To avoid uncertainty in model interpretation, we use an enhanced LIME model with a statistical stability index in this study [22] (https://github.com/giorgiovisani/LIME_stabilitly), which assesses the LIME by developing a complementary pair of indices for stability: the Variable Stability Index (VSI) and the System Stability Index (CSI). The VSI index is used to check whether different LIMEs return the same variables as explanations, and the CSI index controls whether the coefficients of each variable can be considered equal under repeated LIME calls.

**Fig. 4:**
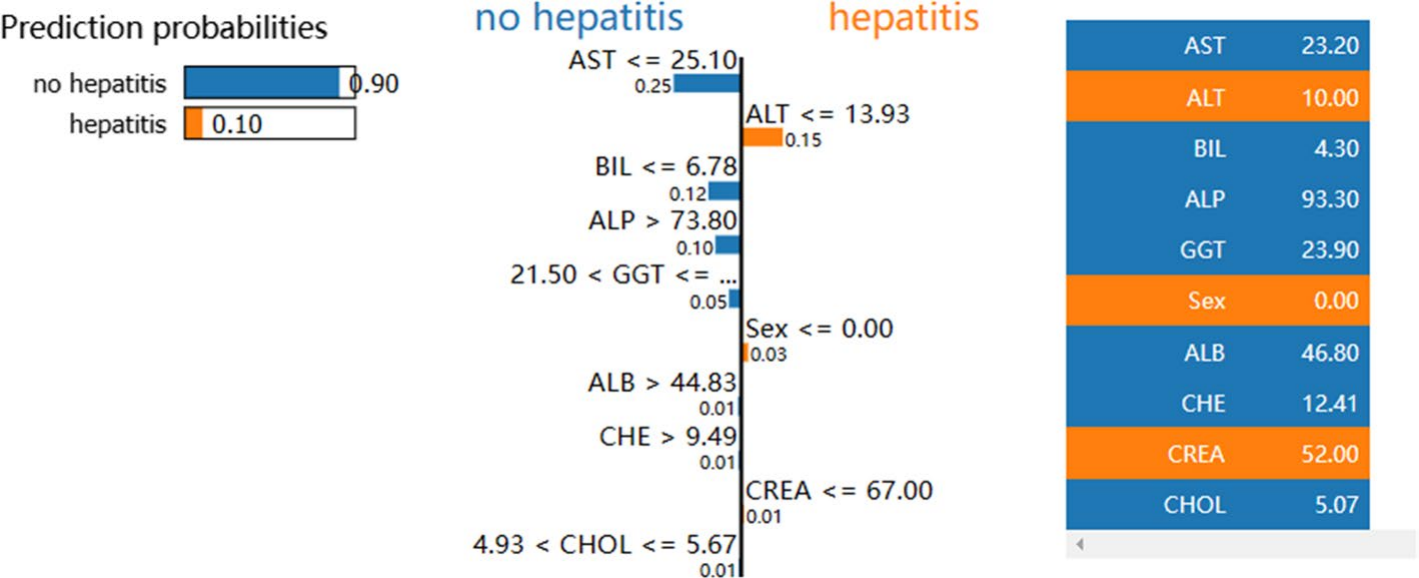
LIME_stabilitly interpretation chart for no hepatitis patients

**Fig. 5:**
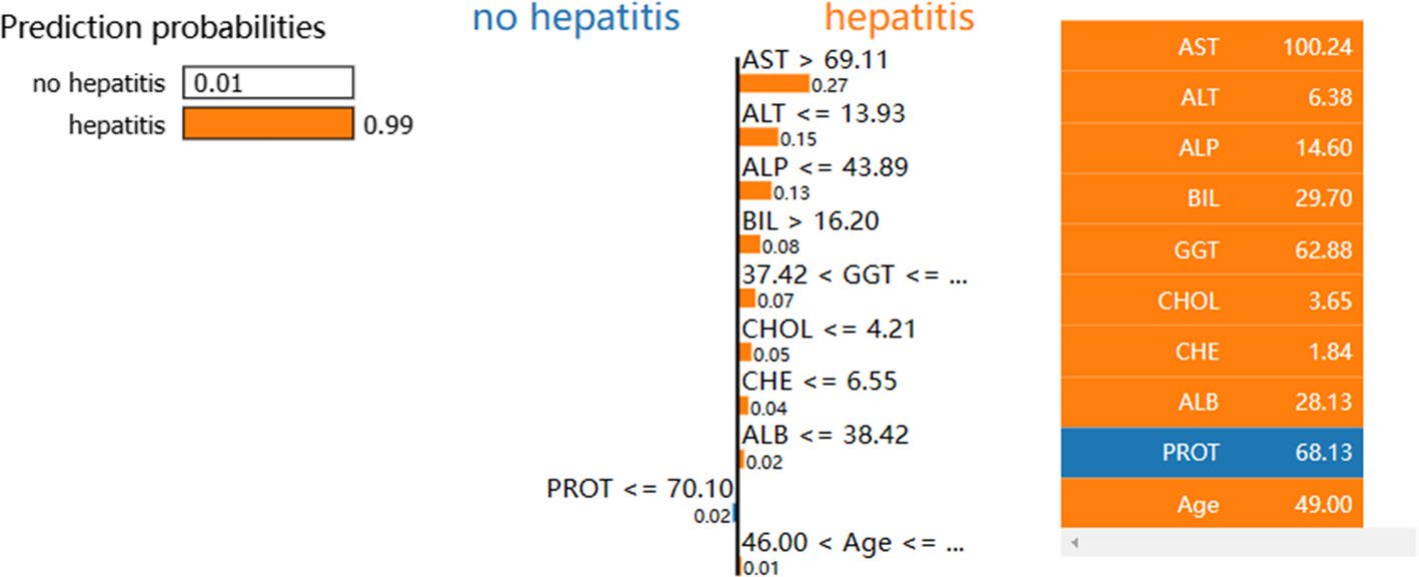
LIME_stabilitly interpretation chart for hepatitis patients

We demonstrate the use of the LIME_stabilitly model on the RF model. As shown in Figs. 4 and 5, the bars on the left represent the contribution of each feature to the no hepatitis class, and the bars on the right depict the contribution of each feature to the prediction of the hepatitis class. Figure 4 indicates that the model has 90% confidence that this patient is a patient without hepatitis, with AST, BIL, ALP, and GGT being the most critical factors. Figure 5 indicates that the model has 99% confidence that this patient is a hepatitis patient, with AST, ALT, ALP, and BIL being the most critical discriminatory factors. It explains the judgment category of individual cases and the basis of discrimination for clinical reference.

### Comparison of IHCP with existing state-of-the-art methods

In this section, we compare the predictive performance of IHCP with existing methods, as shown in Table 5. Akter et al. used machine learning methods to classify normal individuals and patients with hepatitis C. LR performed the best with an accuracy of 95% [23]. Edeh et al. [24] proposed the use of an ensemble learning predictive model to predict patients with hepatitis C. It achieved an accuracy of 95.59%. Safdari et al. proposed a method using SMOTE to eliminate the interclass imbalance and RF as a classifier to get 0.998 AUC and 97.29% accuracy [25]. Li [26] proposed a predictor selection strategy based on a stepwise random forest and logistic regression model combined with the SMOTE technique [26], ultimately achieving an accuracy of 98.74% and an AUC of 0.9401. Yağanoğlu et al. [27] used feature extraction techniques to obtain new features and trained the dataset on multiple classifiers, DT performed the best, obtaining 99.31% accuracy and 0.98 AUC. According to the most recent study to our knowledge, Alizargar et al. [28] proposed a method using XGboost to get 0.984 AUC and 95% accuracy. Compared with the above-proposed method, our proposed IHCP model obtained 99.44% accuracy and 0.9986 AUC, both of which were improved compared with the previous method. Also, our IHCP provides a good global and local interpretation of the prediction results. This allows physicians to better understand the process predicted by the model and integrate it with reality to improve clinical usability.

**Table 5:** Comparison of IHCP with existing art-the-start methods on the UCI dataset

Author	Method	Accuracy (%)	AUC
Akter [23]	LR	95	–
Edeh et al. [24]	Ensemble Learning	95.59	–
Safdari et al. [25]	RF	97.29	0.998
Li [26]	SPFLR	98.74	0.9401
Yağanoğlu [27]	DT with new feature	99.31	0.98
Alizargar et al. [28]	XGBoost	95	0.984
This study	IHCP	**99.44**	**0.9986**

Bold represents the best performing indicator

To demonstrate the superiority and robustness of our proposed model, we conducted experiments on an independent testing set. Huynh et al. [29] proposed an ensemble method that obtained 83.42% accuracy and 0.8418 AUC on this dataset, and Rosly et al. [30] proposed a stacking technique combined with a multilayer perceptron that obtained 86.25% accuracy. In comparison, as shown in Table 6, our IHCP has improved in both AUC and accuracy and is more suitable as a prediction model for hepatitis C.

**Table 6:** Comparison of IHCP with existing art-the-start methods on the independent testing set

Method	Accuracy (%)	AUC
Ensemble learning [29]	83.42	0.8418
Stacking + MLP [30]	86.25	–
IHCP	91.48	0.9895

Based on the cross-validation comparison results and independent testing, we demonstrate the robustness and superior performance of IHCP for the hepatitis prediction problem. At the same time, our model provides interpretability for medical practitioners while ensuring high predictive performance. Among them, the global interpretation provides physicians with which indicators are abnormal causing the disease, and the local interpretation is analyzed for individuals. The interpretability makes the prediction process of the model transparent, allowing medical workers without specialized knowledge to understand the prediction process of the prediction model, and helps accelerate the process of machine learning-based hepatitis prediction models to clinical use.

## Conclusion

In this study, we propose a new computational method IHCP that can perform hepatitis C prediction more accurately and interpretably, SMOTE is used to eliminate the class imbalance in the dataset, Bayesian optimized random forest is selected as the final prediction model, and SHAP and LIME_stabilitly are used to perform interpretative analysis of the model. The experimental results and independent testing show that IHCP obtains significant performance gains compared to the state-of-the-art methods. Notably, IHCP has good interpretability compared to existing methods. This has important applications for improving the diagnosis rate and simplifying the diagnostic process for hepatitis C patients.

However, there are still some limitations that need to be further investigated later. First, more factors need to be considered when deploying the model in real-world scenarios, such as the patient's medical history and lifestyle habits. Second, most clinical scenarios require more information beyond binary prediction. Finally, we hope to use higher-quality datasets to enhance model performance in future studies and to use more interpretable methods to explain the potential predictive patterns of the model.

## Method

### The benchmark dataset

High-quality benchmark datasets are essential for building reliable computational models. The Dataset used in this work was obtained from the publicly available UCI machine learning repository [31]. The multivariate data type includes 615 samples with 13 input attributes and 1 output attribute. These column attributes were: patient ID/number, diagnostic category, age, gender, ALB, ALP, ALT, AST, BIL, CHE, CHOL, CREA, GT, and PROT. The multi-category dataset sample consists of 4 labels (‘0 = blood donor’, ‘0s = suspected blood donor’, ‘1 = hepatitis’, ‘2 = fibrosis’, ‘3 = cirrhosis’). Subsequently, we perform pre-processing and data balancing operations on the raw data to further construct the prediction model.

To validate the generalization capability of the proposed model, we conducted independent dataset tests based on the second dataset. This dataset was obtained from the study by Huynh et al. [29]. It contains 155 data samples with 18 input attributes and 1 output attribute. These column attributes were: age, sex, steroid, antivirals, fatigue, malaise, anorexia, liver_big, liver_firm, spleen_palpable, spiders, ascites, varices, bilirubin, alk_phosphate, sgot, albumin, protime, histology, class.

### Data pre-processing

Pre-processing can help improve data quality and ensure that the data used in building the model are meaningful. Generally, the data pre-processing process includes processing missing values, noise data, and inconsistent data.

In this paper, we constructed a binary classifier to identify whether a patient has hepatitis or not. Thus, we perform the following operations. For the UCI dataset, the first step is to remove the columns that are not relevant for predicting patients, the patient ID/number column. The second step is to replace the data labels. We treated both blood and suspected blood donors as non-diseased with label 0 and treated all three types of hepatitis, fibrosis, and cirrhosis as diseased with label 1. The third step was performed for missing values, and the missing data are shown in Table 7. There are only 31 null values in the given dataset, so we chose the mean-filling method to process them.

**Table 7:** Number of missing values per column in the UCI dataset

Column	NUM
Category	0
Age	0
Sex	0
ALB	1
ALP	18
ALT	1
AST	0
BIL	0
CHE	0
CHOL	10
CREA	0
GGT	0
PROT	1

For the independent testing set, we first transform the attribute names to facilitate understanding and comparison, and the changed attribute names are shown in Table 8. Second, the independent testing set has only two classifications, either die or live, and we assign label 0 to live and label 1 to die. Finally, the independent testing set also has some missing values, and the missing cases are shown in Table 9, and we also take the mean-filling approach to process them.

**Table 8:** Comparison table for changing the names of independent testing set attributes

Original attribute name	Changed attribute name
Sgot	AST
Protime	PROT
Albumin	ALB
alk_phosphate	ALP
Bilirubin	BIL

**Table 9:** Number of missing values per column in the independent testing set

Column	NUM	Column	NUM
Age	0	Sex	0
Steroid	1	Antivirals	0
Fatigue	1	Malaise	1
Anorexia	1	liver_big	10
liver_firm	11	spleen_palpable	5
Spiders	5	Ascites	5
Varices	5	BIL	6
ALP	29	AST	4
ALB	16	PORT	67
Histology	0	Category	0

### Handling imbalanced data

In practice, many datasets are imbalanced. A highly imbalanced dataset will lead to overfitting of the model and further affect the prediction results. Therefore, the operation of balancing the dataset is particularly important to improve the universality and generalization of the model. The main methods to deal with data imbalance are class balancer, resampling, synthetic minority oversampling, and component-sensitive classifier [32]. In this work, dataset balancing is done by resampling, including oversampling and undersampling [33]. Oversampling is the random sampling from the minority category sample to add new samples so that the number of minority category samples is the same as the number of majority category samples. Undersampling is the process of sampling the same number of samples from the majority class sample as the minority class sample.

In this study, the UCI dataset we used was divided into 540 positive samples and 75 negative samples.

**Fig. 6:**
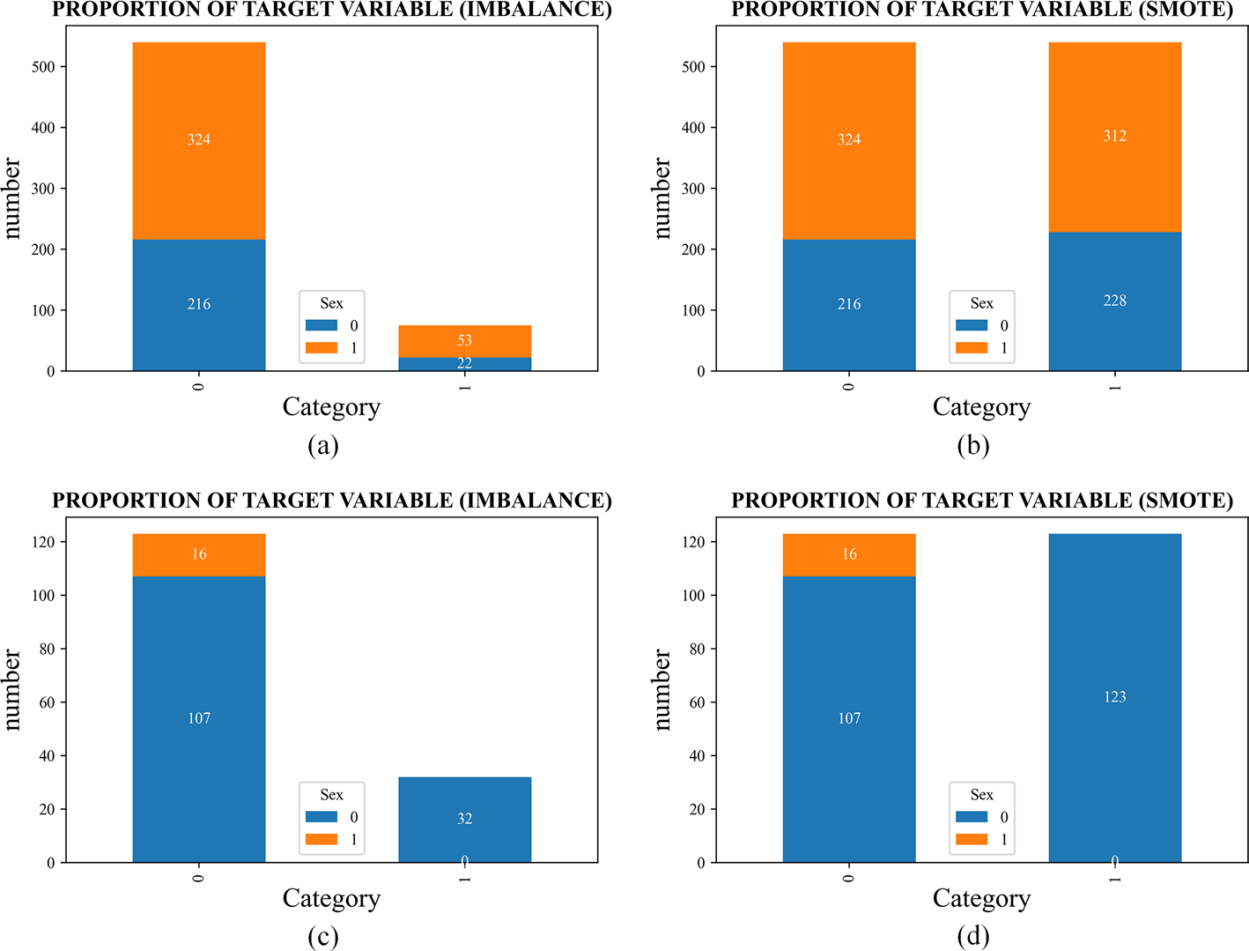
Comparison of datasets before and after balancing. **a** Comparison of the original unbalanced dataset in the UCI dataset. **b** Comparison of the dataset after the oversampling process in the UCI dataset. **c** Comparison of the original unbalanced dataset in the independent testing set. **d** Comparison of the dataset after the oversampling process in the independent testing set. (Sex: 0 = female, 1 = male; category: 0 = no hepatitis, 1 = hepatitis)

Figure 6a shows a bar chart comparing the number of patients with and without liver disease. Since the number of positive and negative samples in the dataset is hugely unbalanced, a simple oversampling technique would result in the divided training set and the testing set containing many duplicate negative samples. Therefore, we use the Synthetic Minority Oversampling Technique (SMOTE) to process the imbalanced data. The bar chart comparing the number of patients with and without liver disease after processing is shown in Fig. 6b, at which time there are 540 positive and 540 negative samples, and the dataset is balanced. We perform the same operation on the independent testing set and compare the independent testing set data before and after balancing, as shown in Fig. 6c, d. Then, the datasets are partitioned into a training set and a testing set in a 4:1 ratio, and the datasets are trained using fivefold cross-validation. When performing cross-validation, first, the dataset is divided into equal quintiles, using the first fold as the testing set and the remaining 2–5 folds as the training set to obtain a prediction accuracy; then, the second fold is used as the testing set, the other first, third, fourth, and fifth folds as the training set, and so on. Finally, five prediction accuracies will be obtained, and the average value will be taken as the final accuracy of the model.

### Model overview

**Fig. 7:**
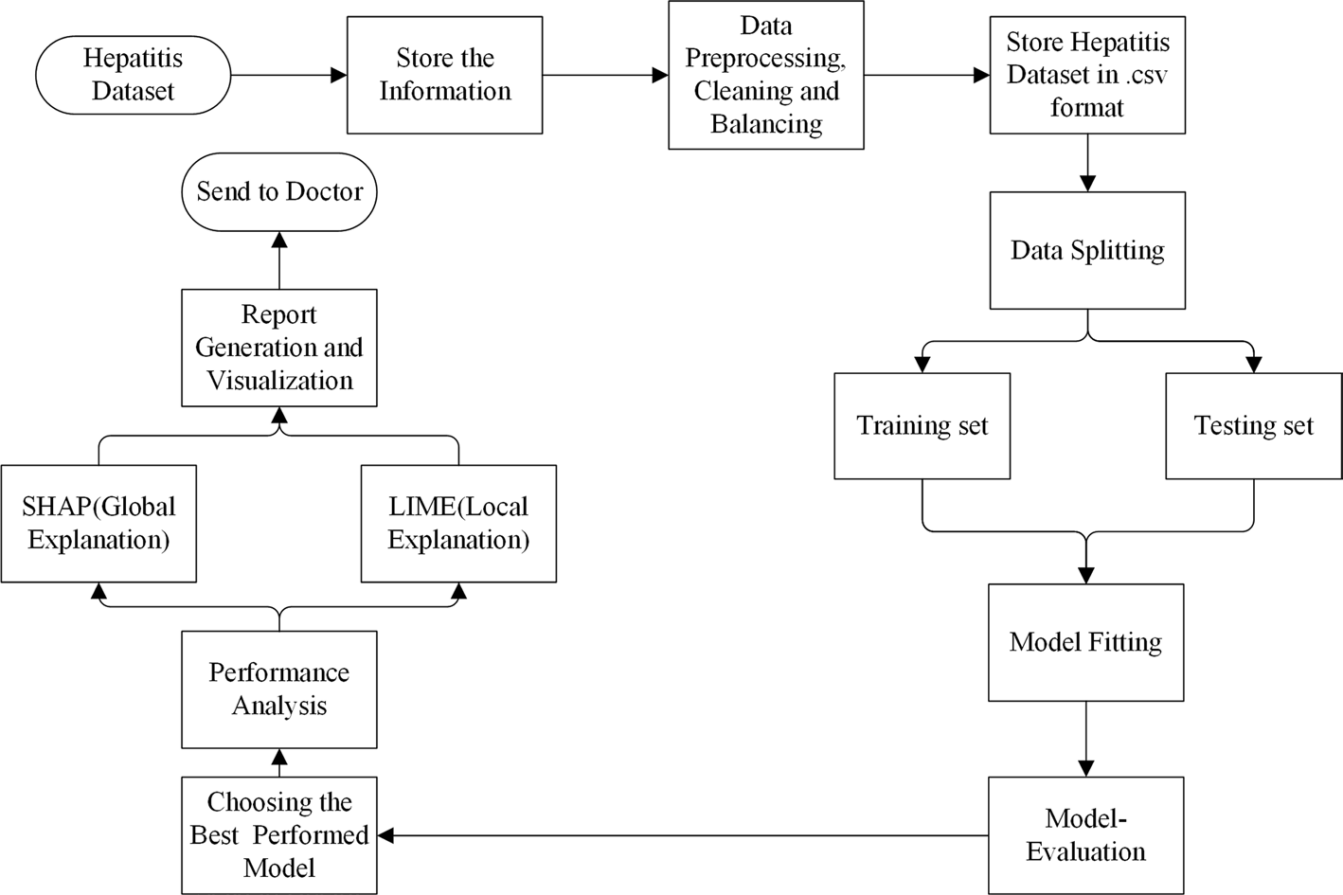
Overview of predicting hepatitis C patients and interpreting model predictions. Collect hepatitis dataset. Preprocess the hepatitis dataset with data cleaning, missing value filling, data balancing, and input into the model. Divide the dataset into training and testing sets to perform training and evaluate the best model. SHAP and LIME are applied to analyze the resulting experimental results

This paper aims to propose an interpretable prediction model for hepatitis. The IHCP framework for predicting hepatitis is shown in Fig. 7. First, the data are pre-processed, which are data cleaning, missing value completion, and data balancing. Then, three different black box models are introduced to train the data. Next, the optimal model was selected using five evaluation criteria. Finally, the models were interpreted globally and locally using visualization methods, and the obtained results were analyzed.

### Classification method

**Fig. 8:**
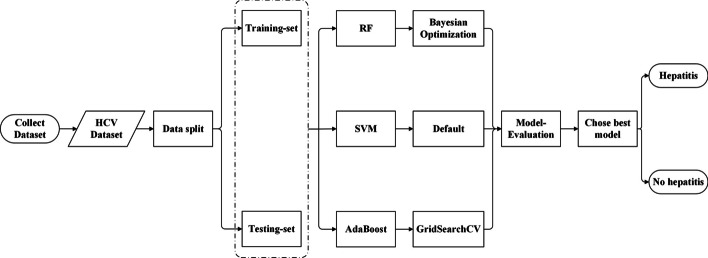
Model processing flowchart

In this section, we describe the three classification methods used by our proposed IHCP for hepatitis identification and the optimization process, and the proposed processing is shown in Fig. 8. Further, we describe the three machine learning methods and the processing in detail.

### Random forest

**Fig. 9:**
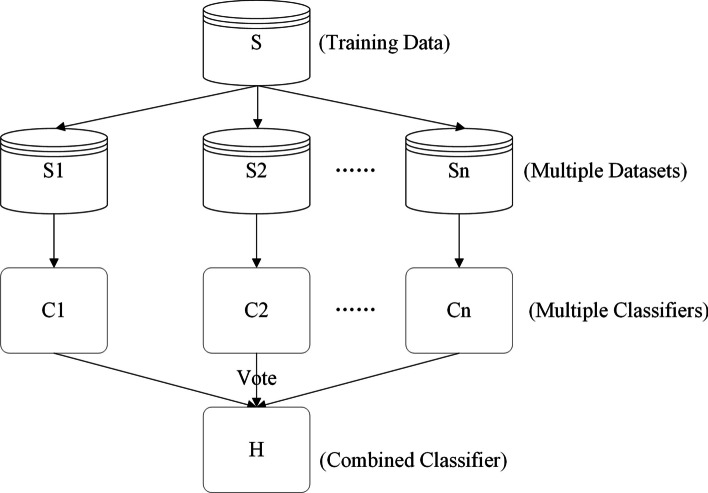
Random forest algorithm workflow

Random Forest is a typical supervised machine-learning method proposed initially by Breiman et al. [34]. It is an algorithm that combines multiple trees through the idea of integration learning, where the basic unit is a decision tree and the integration method used is bagging. The workflow of RF is shown in Fig. 9, RF consists of many decision trees, and each decision tree is a classifier. For any classification sample, N decision trees will have n classification results. The RF uses bagging to integrate these results, using the principle of minority rule to assign the category with the highest number of votes as the final output result. Compared with the decision tree with only one tree, RF solves the disadvantage of the weak generalization ability of the decision tree.

Based on the experimental analysis, we investigated various hyperparameter values of the RF model using a Bayesian optimization approach. It is ensured that the final values used on the validation dataset are the hyperparameters with the highest measurement prediction accuracy. Bayesian optimization is mainly used to solve computationally expensive black-box optimization problems using Bayes' theorem to search for finding the maximum or minimum value of the objective function, which is characterized by using the previously observed prior knowledge at each iteration for the next optimization. Therefore, after constructing the black box model, we used Bayesian optimization to find the optimal RF hyperparameters. We choose the number of iterations to find the optimal parameters to be 30.

### Support vector machine

Support Vector Machines were originally a binary classification model, and the SVM in use today was proposed by Corinna and Vapnik in 1993 [35]. SVM maps the feature vector of an instance to some points in space and classifies the example by drawing a line that best distinguishes these points to classify the cases drawing the line that can best indicate these points [36]; this line is called the maximum interval division hyperplane. This hyperplane allows the algorithm to classify new data more accurately and makes the classifier more robust.

During the experiments, we set default parameter values to train the model. Penalty factor C is set to 1 for higher generalization ability. The kernel function is selected as RBF, and auto is specified as the kernel function coefficient gamma, while probability estimation is enabled.

### AdaBoost

The AdaBoost algorithm is a boosting method proposed initially by Yoav Freund in 1995 [37]. Its core idea is that all samples are given an identical initial weight. A particular feature is selected, and only this feature is used to classify the instances, after which a weak classifier is obtained. Next, a new round of weights is assigned to the samples; misclassified samples are assigned higher weights, and correctly classified samples are assigned lower weights. Then another feature is selected to classify the samples again, and so on. Finally, all the classifiers are weighted and averaged to obtain the final classifier.

When using AdaBoost it is necessary to select the base classifier first. In our experiments, we choose to use the default base classifier, which in general has low complexity. So, we use grid search tuning to tune it. The final experimental results show that the base classifier works better when the number of boosts of the base classifier is chosen to be 50. When the boosting number is too large, it leads to overfitting the model, and too small leads to underfitting the model. Meanwhile, the base classifier has the highest accuracy when the maximum depth max_depth = 3 and the remaining parameter values are not restricted.

### Performance evaluation

Measurement of the performance of the algorithm classification in research is that by using a confusion matrix. We evaluate the proposed model using the following five metrics: accuracy, precision, recall, F1-score, and Area Under Curve (AUC). Higher values for these metrics indicate better performance of the model. The calculation formulas for these metrics are as follows:

1 $${\text{accuracy}} = \frac{{{\text{TP}} + {\text{TN}}}}{{{\text{TP}} + {\text{FP}} + {\text{FN}} + {\text{TN}}}},$$

2 $${\text{precision}} = \frac{{{\text{TP}}}}{{{\text{TP}} + {\text{FP}}}},$$

3 $${\text{recall}} = \frac{{{\text{TP}}}}{{{\text{TP}} + {\text{FN}}}},$$

4 $$ F_{1} - {\text{score }} = \frac{{2\;*\;{\text{precision}}\;*\;{\text{recall}}}}{{{\text{precision}}\; + \;{\text{recall}}}}, $$

where TP represents correctly predicted hepatitis patients, FP represents incorrectly predicted hepatitis patients, TN represents correctly predicted non-hepatitis patients, and FN represents incorrectly predicted non-hepatitis patients.

## Data Availability

The datasets supporting the conclusions of this article are included with article. Project name: IHCP. Project home page: https://github.com/XiqianLu/IHCP. Project inclusion: All datasets and the code needed to replicate the experiment.

## Abbreviations

ALB: Albumin
ALP: Alkaline phosphatase
ALT: Alanine amino transferase
AST: Aspartate amino transferase
BIL: Basic impulse level
CHOL: Cholesterol level
CREA: Creatinine
GGT: Gamma glutamyl tramsferase
PROT: Protime
CHE: Cholinesterase
